# Kaiser Permanente Northern California sepsis mortality reduction initiative

**DOI:** 10.1186/cc11699

**Published:** 2012-11-14

**Authors:** B Crawford, M Skeath, A Whippy

**Affiliations:** 1 Kaiser Permanente, Oakland, CA, USA

## Background

In 2008, Kaiser Permanente Northern California (KPNC), which provides care to 3.3 million members in 21 hospitals, implemented an initiative to improve sepsis care, a critical step to reduce hospital mortality. The goals of the program were threefold: improve identification of sepsis patients, appropriately stratify risk, and reliably provide treatment, focusing on spread and sustainability across all medical centers.

## Methods

In spring 2008, all hospitals reviewed the last 50 deaths and sepsis was identified as a significant improvement opportunity. In May 2008, two hospitals began rapid cycle pilot testing, resulting in the development of a playbook containing treatment algorithms, standardized order sets and flow charts, and chart abstraction tools. These tools, along with expectations for implementation, were shared with leaders and champions from all 21 hospitals at the November 2008 Sepsis Summit. The summit closed with a young mother sharing the story of how her life was saved as a result of the work at the pilot hospital. Subsequently, all hospitals convened multidisciplinary sepsis teams and began training and tool adoption, focusing immediately on improving sepsis identification. Regional mentors and medical center improvement advisors supported team-building and rapid implementation; timely and actionable data allowed ongoing identification of improvement opportunities. Identification and performance monitoring were supported by the development of a web-based tool that pulled information directly from the electronic medical record.

## Results

**Figure 1:**
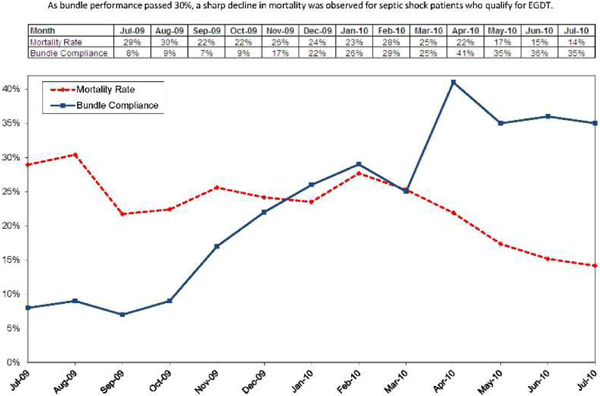
**Sepsis EGDT mortality rate compared with overall bundle compliance**. Kaiser Permanente Northern California Hospitals. As bundle performance passed 30%, a sharp decline in mortality was observed for septic shock patients who qualify for EGDT.

The number of sepsis diagnoses per 1,000 admissions increased from a baseline of 35.7 (March 2008) to 98.3 (December 2010). For septic shock patients, bundle performance increased from 7.3% (Q3 2009) to 55.1% (December 2010), and EGDT population mortality decreased from 29.7% (July to August 2009) to 20.2% (Q4 2010). Overall sepsis mortality decreased from a baseline of 24.6% (March 2008) to 11.5% (December 2010); mortality rates continued to drop to below 9% in May 2012. This was associated with a 14% overall drop in raw hospital mortality. Subsequent performance improvement programs encompass care of the intermediate lactate population, pediatric patients and surgical patients. See Figure 1 and Table 1.

**Table 1:** Key results of the KPNC sepsis mortality reduction initiative (aggregate data for all 21 hospitals)

Measure	Baseline	Rapid adoption (December 2010)^a^	Sustainability (May 2012)^a^
Sepsis diagnoses per 1,000 admits	35.7 (2006 to early 2008)	98.3	137.9
Admitted patients with blood cultures in ED have lactate test in ED	27% (early 2008)	96.5%	95.6%
ABX w/in 60 minutes of dx of shock	69.5% (Q3 2009)	90.4%	91.8%
CL w/in 2 hours of dx (first CVP of ScvO2 in 2 hours)	41.5% (Q3 2009)	78.6%	89.6%
Mean BP (MAP) at target	52% (Q3 2009)	90.4%	93.8%
CVP at target	41.5% (Q3 2009)	83.8%	92.8%
ScvO_2 _at target	30.8% (Q3 2009)	74.3%	81.4%
Lactate lower within 6 hours for EGDT	52% (Q3 2009)	91.2%	95.9%
EGDT bundle	7.3% (Q3 2009)	55.1%	70.1%
Sepsis raw mortality	24.6% (2006 to early 2008)	11.5%	8.7%
Sepsis observed/expected (O/E) mortality	1.07 (rolling year ending Q1 2008)	0.82	0.56
Sepsis O/E LOS	1.07 (rolling year ending Q1 2008)	0.89	0.75
EGDT population mortality (only patients with refractory shock or lactate ≥4)	29.7% (239 cases, July to August 2009)	20.2% (391 cases, Q4 2010)	18.6% (323 cases, March-May 2012)
Raw all cause adult non-OB KPNC hospital mortality	3.63% (2006 to 2007)	3.11% (2010)	3.07% (year ending Q2 2012)
HSMR-Medicate only	0.92 (rolling year ending Q2 2008)	0.60 (YE 2010)	0.52 (YE 2011)
Balancing measures: EGDT associated harm	**July to December 2009**	**July to December 2010**	**December 2011 to May 2012**
BSI	0	0	0
Retained guidewires	3	0	0
Pneumothorax	1	2	4

^a^Rapid adoption and sustainability data reflect December 2010 and May 2012, respectively, unless otherwise noted.

## Conclusion

The KPNC program is unique in its rapid rate of improvement in sepsis measures, adoption of a single standard of care across an entire 21-hospital system, sustainability well beyond the rapid adoption period, and the quantification of mortality risk beyond the shock population to the intermediate sepsis population. These results demonstrate that a strong performance improvement engine can drive large-scale, sustained improvements in care within a short duration.

